# T-cell subsets and cytokines are indicative of neoadjuvant chemoimmunotherapy responses in NSCLC

**DOI:** 10.1007/s00262-024-03687-5

**Published:** 2024-04-15

**Authors:** Ling Yi, Ziwei Xu, Tianyu Ma, Chong Wang, Panjian Wei, Bo Xiao, Hongtao Zhang, Nanying Che, Zhidong Liu, Yi Han

**Affiliations:** 1 Department of Central Laboratory, Beijing Tuberculosis and Thoracic Tumor Research Institute, Beijing Chest Hospital, Capital Medical University, Beijing, China; 2 Department of Minimally Invasive Surgery, Beijing Tuberculosis and Thoracic Tumor Research Institute, Beijing Chest Hospital, Capital Medical University, Beijing, China; 3 Department of Thoracic Surgery II, Beijing Tuberculosis and Thoracic Tumor Research Institute, Beijing Chest Hospital, Capital Medical University, Beijing, China; 4 Department of Pathology, Beijing Tuberculosis and Thoracic Tumor Research Institute, Beijing Chest Hospital, Capital Medical University, Beijing, China

**Keywords:** Neoadjuvant chemoimmunotherapy, Predictive markers, NSCLC, CD8 + T cells, Tregs, Cytokines

## Abstract

**Purpose:**

Neoadjuvant PD-1 blockade combined with chemotherapy is a promising treatment for resectable non-small cell lung cancer (NSCLC), yet the immunological mechanisms contributing to tumor regression and biomarkers corresponding to different pathological responses remain unclear.

**Methods:**

Using dynamic and paired blood samples from NSCLC patients receiving neoadjuvant chemoimmunotherapy, we analyzed the frequencies of CD8 + T-cell and Treg subsets and their dynamic changes during neoadjuvant treatment through flow cytometry. Cytokine profiles and function-related gene expression of CD8 + T cells and Tregs were analyzed through flow cytometry and mRNA-seq. Infiltrating T-cell subsets in resected tissues from patients with different pathological responses were analyzed through multiplex immunofluorescence.

**Results:**

Forty-two NSCLC patients receiving neoadjuvant chemoimmunotherapy were enrolled and then underwent surgical resection and pathological evaluation. Nineteen patients had pCR (45%), 7 patients had MPR (17%), and 16 patients had non-MPR (38%). In patients with pCR, the frequencies of CD137 + CD8 + T cells (*P* = 0.0475), PD-1 + Ki-67 + CD8 + T cells (*P* = 0.0261) and Tregs (*P* = 0.0317) were significantly different from those of non-pCR patients before treatment. pCR patients usually had low frequencies of CD137 + CD8 + T cells, PD-1 + Ki-67 + CD8 + T cells and Tregs, and their AUCs were higher than that of tissue PD-L1 expression. Neoadjuvant chemoimmunotherapy markedly improved CD8 + T-cell proliferation and activation, especially in pCR patients, as the frequencies of CD137 + CD8 + (*P* = 0.0136) and Ki-67 + CD8 + (*P* = 0.0391) T cells were significantly increased. The blood levels of cytokines such as IL-2 (*P* = 0.0391) and CXCL10 (*P* = 0.0195) were also significantly increased in the pCR group, which is consistent with the high density of activated cytotoxic T cells at the tumor site (*P* < 0.0001).

**Conclusion:**

Neoadjuvant chemoimmunotherapy drives CD8 + T cells toward a proliferative and active profile. The frequencies of CD137 + CD8 + T cells, PD-1 + Ki-67 + CD8 + T cells and Tregs at baseline might predict the response to neoadjuvant chemoimmunotherapy in NSCLC patients. The increase in IL-2 and CXCL10 might reflect the chemotaxis and enrichment of cytotoxic T cells at the tumor site and a better response to neoadjuvant chemoimmunotherapy.

**Supplementary Information:**

The online version contains supplementary material available at 10.1007/s00262-024-03687-5.

## Introduction

Lung cancer is the leading cause of cancer-related death worldwide. Non-small cell lung cancer (NSCLC) accounts for approximately 80–85% of newly diagnosed lung cancer cases annually [1–3]. Surgical resection with curative intent remains the mainstay treatment for early-stage NSCLC; however, its 5-year survival rates remain unsatisfactory due to the high rates of recurrence and metastasis [3, 4]. Patients with resectable NSCLC are often treated with surgery and adjuvant chemotherapy [5]. Nevertheless, these patients still have a high risk of recurrence and death [6]. Additionally, little progress has been made in the treatment of resectable NSCLC over the past several decades [7].

Neoadjuvant immunotherapy is a promising approach for improving long-term survival and increasing the cure rates for patients with early-stage lung cancer [8]. Immune checkpoint blockade (ICB) targeting the PD-1/PD-L1 axis has demonstrated remarkable therapeutic efficacy against advanced NSCLC and may shed new light on potential therapeutic breakthroughs. Ongoing trials evaluating these agents are rapidly moving from advanced NSCLC to earlier-stage disease and from palliative to curative intent [7, 9]. Neoadjuvant immunotherapies are evolving rapidly, and new clinical trial data and biological discoveries are continuously being reported [3]. At the 2023 ASCO Annual Meeting, multiple phase III clinical trials, such as AEGEAN, Neotorch and KEYNOTE-671, were presented, aiming to further demonstrate the clinical efficacy of various PD-1 inhibitors, such as durvalumab, toripalimab and pembrolizumab [10–12]. Some clinical trials adopt perioperative strategies in which PD-1 inhibitors are combined with chemotherapy for 4 cycles during the neoadjuvant treatment stage, and then, maintenance therapy with 13 cycles of PD-1 inhibitors after surgery is administered. Interestingly, Neotorch has pioneered the ‘3 + 1 + 13’ perioperative treatment model with 3 cycles of chemoimmunotherapy before surgery + 1 cycle of chemoimmunotherapy after surgery + 13 cycles of immunotherapy maintenance, marking the true implementation of neoadjuvant chemoimmunotherapy in the perioperative period. Moreover, studies indicate that neoadjuvant chemoimmunotherapy may significantly improve the rates of MPR or pCR, reduce systemic recurrence and extend EFS in patients with resectable NSCLC [8, 13, 14]. Nevertheless, there are several unresolved challenges, and chemoimmunotherapy fails in more than half of treated patients. Thus, the identification of biomarkers for predicting clinical efficacy is an urgent issue.

T cells are key determinants of the immune response to checkpoint blockade [15]. In addition to PD-L1 expression, CD8 T cells distinctly at the tumor margin [16], the presence of PD-1 + CD8 + T cells in tumors [17] and high densities of CD45 + T cells have been linked to a clinical benefit from PD-1 blockade based on pretreatment tumor specimens [18]. However, blockade of PD-1 signaling with anti-PD-1/PD-L1 antibodies not only reinvigorates preexisting tumor-specific T cells but also augments PD-1 + Tregs; therefore, the balance of PD-1 expression between CD8 + T cells and Treg cells in the TME needs more attention [19].

Although local tissue-based immune responses are critical for elucidating direct tumor–immune cell interactions, this procedure is invasive and time-consuming. Thus, faster, less invasive and more sensitive tools are needed to detect useful molecular and/or clinical predictors of treatment response [20, 21]. In this study, we evaluated serial blood samples from patients with resectable NSCLC undergoing standard neoadjuvant chemoimmunotherapy to characterize the evolution of peripheral immune cells, such as CD8 + T-cell and Treg subsets, and preliminarily described the possible mechanisms of different pathological responses in NSCLC patients.

## Methods

### Patients and sample collection

NSCLC patients who were pathologically diagnosed; had local lymph node metastases or locally advanced disease, without evidence of distant metastases; had no EGFR, ALK or ROS1 mutations; were previously untreated; and were going to receive neoadjuvant treatment were recruited. Patients with another diagnosed tumor in the previous 10 years; with inflammatory diseases, such as TB or pneumonia; with a life expectancy of ≤ 6 months; receiving immunosuppressive medications; with a history of organ allograft; or without clinicopathological evaluation were excluded. From October 2020 to December 2022, 42 patients receiving neoadjuvant chemoimmunotherapy and surgical resection of primary tumors at the Department of Thoracic Surgery, Beijing Chest Hospital, were enrolled. Among the 42 patients, peripheral blood samples were collected from 23 patients at the time of diagnosis or 1–2 days before administration and before surgery. Plasma samples and peripheral blood mononuclear cells (PBMCs) were stored at −80 °C and in liquid nitrogen. Resection tissue samples (formalin-fixed, paraffin-embedded, FFPE sections) from these 42 patients were also collected. All detailed clinical patient data are summarized in Supplementary Tables 1 and 2. This research was approved by the ethics committee of Beijing Chest Hospital (YJS-2021-038), and all patients signed an informed consent form.

### Response evaluation

For the purpose of tumor response evaluation (modified WHO criteria), radiological evaluations were conducted at baseline and at every re-examination. The treatment response was classified as complete response (CR), partial response (PR), stable disease (SD) and progressive disease (PD) according to the Response Evaluation Criteria in Solid Tumors (RECIST version 1.1) [22], and monitoring was continued in all treated patients. The pathological response was assessed by professional pathologists at Beijing Chest Hospital. Pathological complete response (pCR) was defined as 0% viable tumor cells in the residual tumor, and tumors with < 10% viable tumor cells were considered to have a major pathological response (MPR) according to hematoxylin and eosin staining.

### Multiparameter flow cytometry

Blood T-cell subsets from 23 patients were analyzed by flow cytometry. First, patient peripheral blood was lysed (lysing buffer, BD Biosciences), and Fc was blocked (BD Biosciences). Then, CD8 + T-cell and Treg subsets were identified with a panel of antibodies: BV-510-anti-CD3 (HIT3a), APC-H7-anti-CD4 (RPA-T4), BUV395-anti-CD8 (RPA-T8), BB515-anti-CD38 (HIT2), BV786-anti-CD45RO (UCHL1), PE-anti-CD137 (4B4-1) and AF-647-anti-PD-1 (NAT105, BioLegend). After extracellular staining, the cells were fixed and permeabilized with BB700-anti-FOXP3 (236A/E7), BV650-anti-IFN-γ (4S.B3) and PE-Cy7-anti-Ki-67 (B56) according to the manufacturer’s protocol. The stained cells were washed, resuspended and analyzed with an LSRFortessa flow cytometer (BD Biosciences). All antibodies used for flow cytometry analysis except anti-PD-1 were purchased from BD Biosciences.

To isolate CD8 + T cells and Tregs for RNA sequencing (RNA-seq), patient PBMCs were stained with PerCP-anti-CD3 (SK7), PE-Cy7-anti-CD4 (RPA-T4), APC-H7-anti-CD8 (SK1), BB515-anti-CD25 (2A3), AF-647-anti-CD127 (HIL-7R- M21) and 7-AAD (BD Pharmingen), and then, the resuspended cells were sorted with a FACSAria III flow cytometer. All antibodies used were purchased from BD Biosciences.

### RNA sequencing of T-cell subsets

For RNA-seq library preparation and sequencing, total RNA was extracted from CD8 + T cells and Tregs using the RNeasy Micro Kit (QIAGEN GmbH). Total RNA was sent to the Beijing Genomics Institute for RNA quantity and purity assessment and library construction following standard protocols. The library products were sequenced using an MGISEQ-2000 instrument (BGI-Shenzhen, China). Standard bioinformatic analysis was performed by the Beijing Genomics Institute.

### Multiplexed quantitative immunofluorescence

After neoadjuvant treatment, tumor-infiltrating lymphocyte (TIL) subtypes were assessed in situ by multiplexed quantitative immunofluorescence (QIF) on formalin-fixed, paraffin-embedded (FFPE) sections using the PANO 6-Plex IHC Kit (Panovue, Beijing, China). In brief, histological sections from the patients were deparaffinized and subjected to antigen retrieval; the sections were then stained with antibodies against pan-CKs (ab27988, clone AE1/AE3, 1:500 dilution), CD8 (ab17147, clone C8/144B, 1:200), Foxp3 (ab20034, clone 236A/E7, 1:200) and PD-1 (ab52587, NAT105, 1:50). The stained slides were scanned with a Vectra multispectral microscope (Akoya Biosciences). Images of single-color-stained tissue sections were obtained, and the spectrum of each fluorophore was extracted to create the needed spectrum for multiplex immunohistochemical staining, which was performed in InForm2.4.8 (Akoya Biosciences). The densities of cytotoxic T cells (CD8 +), PD-1 + cytotoxic T cells (PD-1 + CD8 +), Tregs (FOXP3 +) and PD-1 + Tregs (PD-1 + FOXP3 +) were calculated, and the data are presented as the percentage of positive cells among total cells in each selected field. The stained slides were visually examined by a pathologist.

### Blood cytokine analysis

Three milliliters of whole blood (3 ml) was collected using EDTA vacutainer tubes (BD Biosciences) and centrifuged at 3000 rpm for 10 min at 4 °C. Then, plasma was collected and stored at −80 °C. All plasma samples were thawed at the same time, and the concentrations of IL-1β, Il-2, IL-4, IL-6, IL-8, IL-10, IL-12p70, CXCL10 (IP-10), TNF-ɑ and IFN-γ in blood were detected using the LEGENDplex™ HU Essential Immune Response Panel (BioLegend) according to the manufacturer’s instructions. In brief, 25 µl of plasma (diluted twofold with assay buffer) for each sample or standard and a mixture of color-coded magnetic beads coated with capture antibodies that recognize specific analytes were first added to 96-well plates and incubated for 2 h on a shaker (800 rpm) at room temperature. Later, biotinylated detection antibodies that bind the analytes of interest were added and incubated for 1 h on a shaker (800 rpm) at room temperature. Then, PE-conjugated streptavidin was added and incubated for 30 min at room temperature. The plates were then washed, and the cytokine concentrations were analyzed using an LSRFortessa instrument.

### Statistical analysis

Statistical analysis was performed using GraphPad Prism 9.0 and SPSS 23.0. Categorical variables are summarized as frequency counts, and measurement data are summarized as the medians and interquartile ranges (IQRs). Measurement data for continuous variables are summarized as the mean and standard deviation. The Chi-square test or Fisher’s exact test was used to analyze the correlation between clinical characteristics and pathological response. The Mann–Whitney test (for non-normally distributed data) and unpaired *t* test (for normally distributed data) were used to compare the proportions of T-cell subsets and blood cytokines between pCR and non-pCR patients; the Wilcoxon matched-pairs signed-rank test (for non-normally distributed data) and paired *t* test (for normally distributed data) were used to assess dynamic changes in cell subsets and blood cytokines before and after neoadjuvant chemoimmunotherapy. *P* < 0.05 was considered statistically significant.

## Results

### Patient demographics and clinical characteristics

A total of 42 NSCLC patients were recruited according to the inclusion and exclusion criteria. All patients received 2–3 cycles of neoadjuvant chemoimmunotherapy and underwent complete lesion resection and pathological evaluation. The trial schema and relevant sample collection contents are summarized in Supplementary Fig. 1. The median patient age was 65.5 years (58–68), 90% (38/42) of patients were male, 81% (34/42) of patients had squamous cell carcinoma, 73.8% (31/42) of patients had a smoking history, and 69% (29/42) of patients had baseline tissue PD-L1 data. Of the 42 patients, 71.4% (30/42) underwent lobectomy, 14.3% (6/42) underwent double lobectomy, and 14.3% (6/42) underwent sleeve resection/bronchoplasty. A total of 38% (16/42) of patients underwent video-assisted thoracoscopic surgery (VATS), and 62% (26/42) underwent thoracotomy. All 42 patients underwent complete resection (R0). The median operation time was 130 min (109–180 min), the median blood loss was 150 ml (100–300 ml), and the median hospital stay in the ICU was 1 day (0–2 days). These data are summarized in Table 1.

**Table 1:** Surgical procedures for NSCLC patients treated with neoadjuvant chemoimmunotherapy

Characteristic	Result
*Extent of surgery (%)*	
Lobectomy	71.42(30/42)
Bilobectomy	14.29 (6/42)
Sleeve resection/bronchoplasty	14.29 (6/42)
*Surgical approach (%)*	
VATS	38.10 (16/42)
Thoracotomy	61.90 (26/42)
*Perioperative outcomes*	
Operation time (min), median (IQR)	130 (109.5–180.3)
Blood loss (mL), median (IQR)	150 (100–300)
Volume of drainage (mL), median (IQR)	1285 (852.5–1870)
Chest tube duration (days), median (IQR)	5 (4–7)
Postoperative length of stay (days), median (IQR)	8 (6–11)
ICU LOS (days), median (IQR)	1 (0–2)

VATS: video-assisted thoracic surgery; FEV1/FVC: forced expiratory volume in the first second/forced vital capacity, which indicates pulmonary ventilation function index; ICU: intensive care unit; LOS: length of stay; IQR: interquartile range

Forty-two patients were divided into two groups according to whether their primary tumor cells had been cleared: the pathological complete response (pCR) and non-pCR groups. Nineteen patients belonged to the pCR group (45%), 7 patients had a major pathological response (MPR) (17%), and 16 patients had non-MPR (38%) and belonged to the non-pCR group (55%). There was no correlation between patient treatment efficacy and age, sex, smoking status, clinical stage, tissue type, tissue PD-L1 expression, prescribed checkpoint inhibitor, number of neoadjuvant therapy cycles or RECIST status (Table 2).

**Table 2:** Baseline characteristics of NSCLC patients treated with neoadjuvant chemoimmunotherapy

Characteristic	Group	All patients	pCR	non-pCR	*P* value
Age (years)					0.2607
	≤ 60	16	9	7	
	> 60	26	10	16	
Sex					0.6135
	Male	38	18	20	
	Female	4	1	3	
Smoking history					> 0.9999
	Never smoker	11	5	6	
	Current/ex-smoker	31	14	17	
Clinical T stage					0.3523
	T1 + T2	22	8	14	
	T3 + T4	20	11	9	
Clinical N stage					0.2092
	N0 + N1	17	10	7	
	N2	25	9	16	
cTNM#					0.3227
	IIA-IIB	12	7	5	
	IIIA-IIIB	30	12	18	
Histology					0.7092
	Adenocarcinoma	8	3	5	
	Squamous cell carcinoma	34	16	18	
PD-L1 expression					0.9731
	< 1%	3	1	2	
	1–49%	7	3	4	
	≥ 50%	19	9	10	
	NA	13	6	7	
Prescribed checkpoint inhibitor					0.1577
	Camrelizumab	29	15	14	
	Sintilimab	8	3	5	
	Tislelizumab	4	0	4	
	Pembrolizumab	1	1	0	
Neoadjuvant therapy cycles					> 0.9999
	2	39	18	21	
	3	3	1	2	
RECIST status					0.5301
	PR	26	13	13	
	SD	16	6	10	

NA: not applicable

### Baseline peripheral blood CD8 + T-cell subsets are indicative of treatment response

**Fig. 1:**
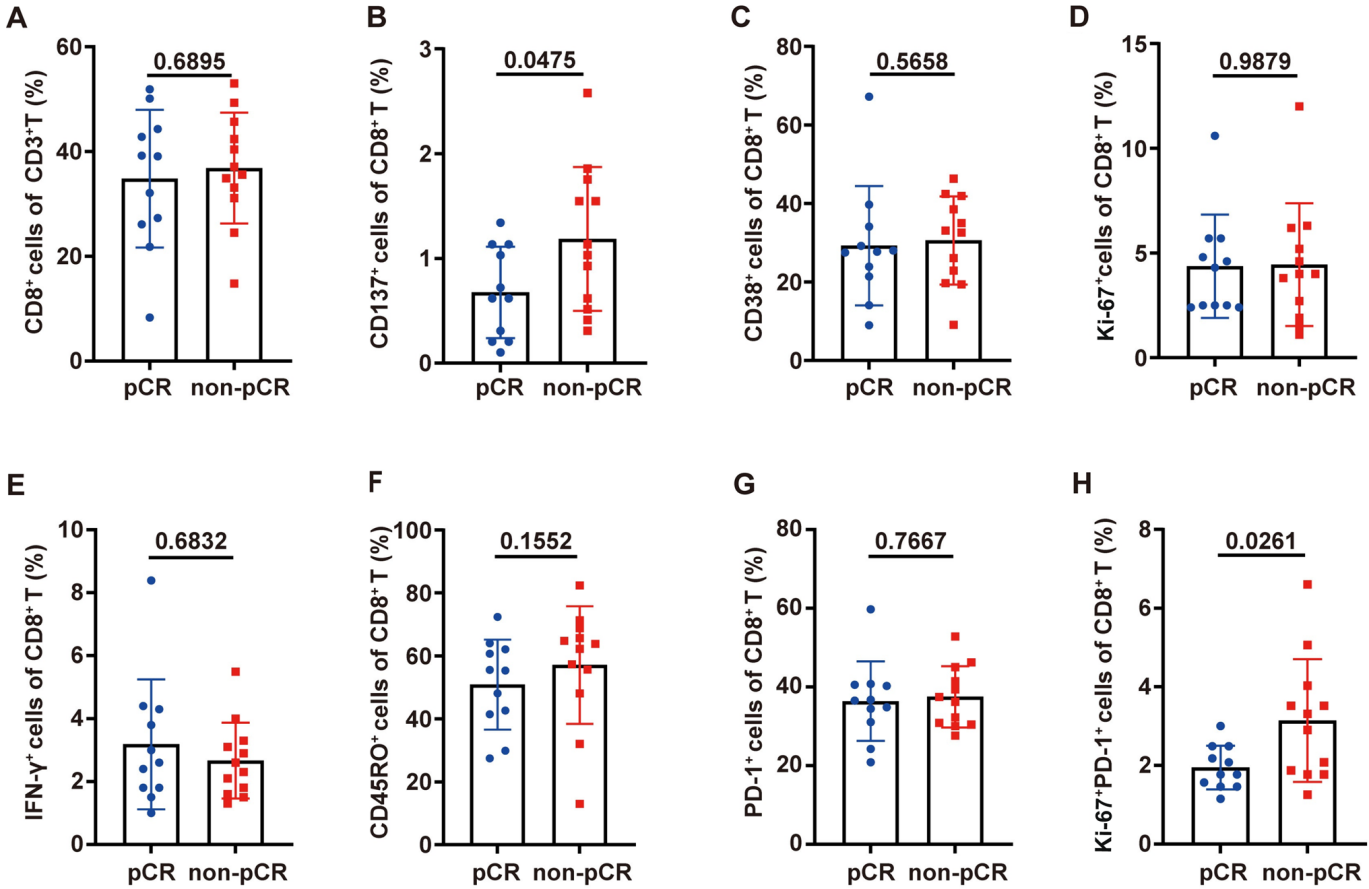
Baseline CD8 + T subsets were correlated with the response to neoadjuvant chemoimmunotherapy in NSCLC patients. Blood CD8 + T-cell subsets from 23 patients were analyzed before treatment. The patients were divided into a pCR group (*n* = 11) and a non-pCR group (*n* = 12) according to their pathological evaluation after surgery, and the differences in their CD8 + T-cell subset frequencies were compared. Comparison of the frequencies of CD8 + T cells (**A**, percentage of CD8 + T cells/CD3 + T cells, unpaired *t* test), CD137 + CD8 + T cells (**B**, percentage of CD137 + CD8 + T cells/CD8 + T cells, unpaired *t* test), CD38 + CD8 + T cells (**C**, Mann–Whitney test), Ki-67 + CD8 + T cells (**D**, Mann–Whitney test), IFN-γ + CD8 + T cells (**E**, Mann–Whitney test), CD45RO + CD8 + T cells (**F**, Mann–Whitney test), PD-1 + CD8 + T cells (**G**, unpaired *t* test) and PD-1 + Ki-67 + CD8 + T cells (**H**, unpaired *t* test) between pCR and non-pCR patients

Baseline peripheral blood samples were collected and analyzed for 23 out of 42 patients. First, we analyzed the frequency of CD8 + T-cell subsets (Supplementary Table 3) in the peripheral blood to determine whether these subsets were associated with treatment response. The mean frequencies of CD8 + T cells (CD8 + T cells/CD3 + T cells, normal distribution, mean ± SD) were 34.82% ± 13.15% and 36.83% ± 10.57% in the pCR group (*n* = 11) and non-pCR group (*n* = 12), respectively, before treatment, and there was no difference in the CD8 + T-cell frequency between the pCR and non-pCR groups (unpaired *t* test, *P* = 0.6895, Fig. 1A). Then, CD8 + T cells were further subdivided into CD137 + CD8 + T-cell, CD38 + CD8 + T-cell, Ki-67 + CD8 + T-cell, IFN-  γ+ CD8 + T-cell, CD45RO + CD8 + T-cell, PD-1 + CD8 + T-cell and PD-1 + Ki-67 + CD8 + T-cell subsets, and the gating strategy for these CD8 + T-cell subsets is shown in Supplementary Fig. 2A, B. The mean CD137 + CD8 + T-cell percentages (CD137 + CD8 + T cells/CD8 + T cells, normal distribution, mean ± SD) in the pCR and non-pCR groups were 0.65% ± 0.42% and 1.15% ± 0.67%, respectively, and the baseline CD137 + CD8 + T-cell percentage in the non-pCR group was significantly higher than that in the pCR group (unpaired *t* test, *P* = 0.0475, Fig. 1B). The median frequencies of CD38 + CD8 + T cells, Ki-67 +  CD8 + T cells, IFN-γ + CD8 + T cells and CD45RO + CD8 + T cells, and the mean frequencies of PD-1 + CD8 + T cells were not significantly different between pCR and non-pCR patients (Fig. 1C–G). The mean frequencies of PD-1 + Ki-67 + CD8 + T cells (PD-1 + Ki-67 + CD8 + T cells/CD8 + T cells, normal distribution, mean ± SD) were 1.87% ± 0.54% and 3.03% ± 1.52%, respectively, and the baseline PD-1 + Ki-67 + CD8 + T-cell percentage in the non-pCR group was also significantly higher than that in the pCR group (unpaired *t* test, *P* = 0.0261, Fig. 1H).

### Baseline peripheral blood Treg subsets are indicative of treatment response

**Fig. 2:**
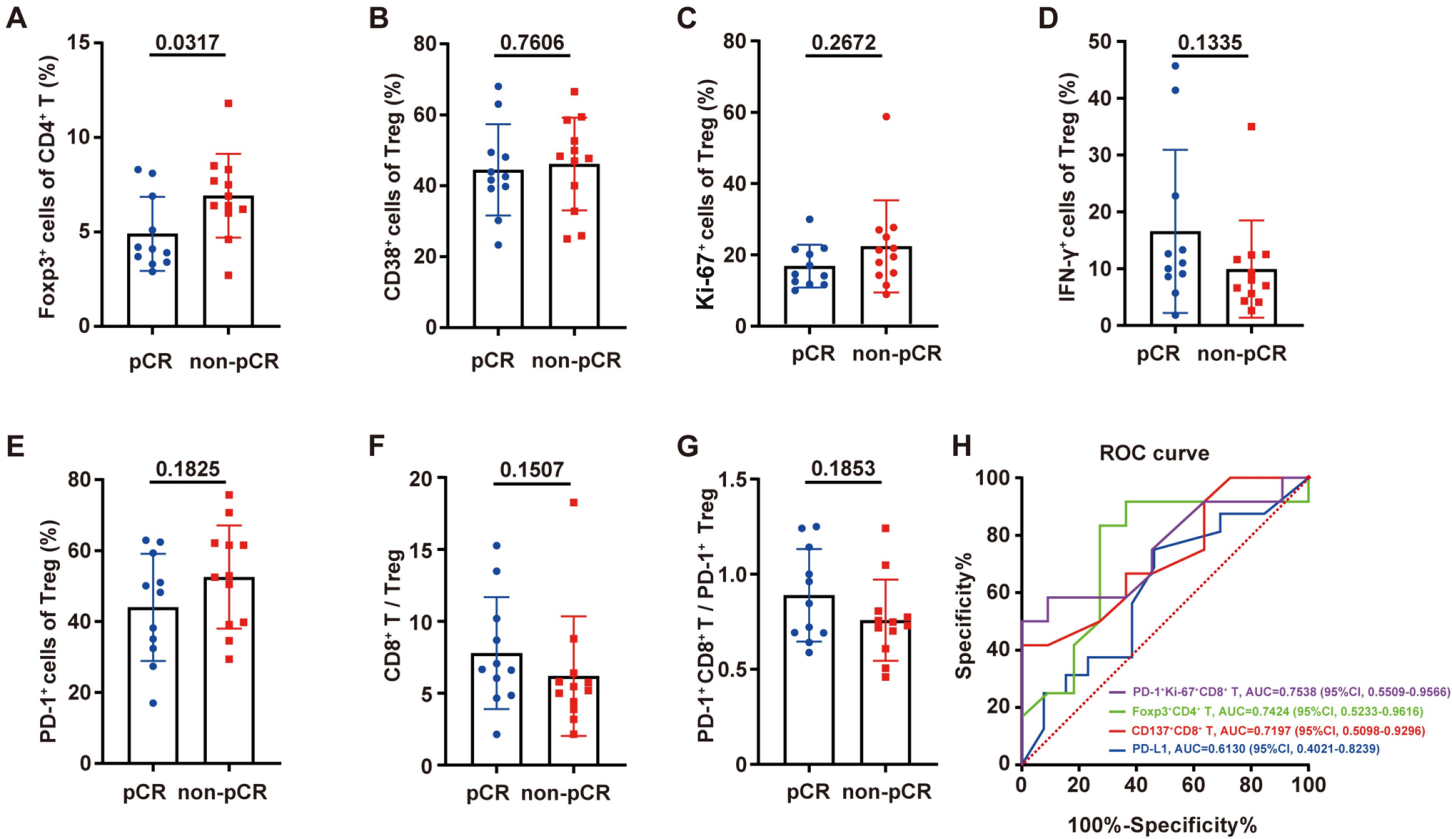
Comparison of the frequency of baseline Treg subsets and the AUCs of different indicators. The blood Treg subsets for 23 patients were analyzed and compared before treatment. The difference in the frequencies of Treg cells (**A**, percentage of CD4 + FOXP3 + cells/CD4 + T cells, unpaired *t* test), CD38 + Tregs (**B**, unpaired *t* test), Ki-67 + Tregs (**C**, Mann–Whitney test), IFN-γ + Tregs (**D**, Mann–Whitney test) and PD-1 + Tregs (**E**, percentage of PD-1 + CD4 + FOXP3 + T cells/CD4 + FOXP3 + T cells, unpaired *t* test) at baseline between pCR and non-pCR patients. The ratio of CD8 + T cells/Tregs (**F**, Mann–Whitney test) and PD-1 + CD8 + T cells/PD-1 + Tregs (**G**, unpaired *t* test) was also compared between pCR and non-pCR patients. **H**, Comparison of the areas under the ROC curves (AUCs) of the CD137 + CD8 + T-cell percentage (red), PD-1 + Ki-67 + CD8 + T-cell percentage (purple), Treg percentage (green) and tissue PD-L1 expression (*n* = 29, blue) between pCR and non-pCR patients

We identified cells coexpressing CD4 and FOXP3 as Tregs in flow cytometry analysis and analyzed the frequency of Treg subsets (Supplementary Table 3) in the peripheral blood of 23 patients to determine whether there were differences in Treg subsets in patients with different therapeutic responses. The mean Treg percentages (CD4 + FOXP3 + cells/CD4 + T cells, normal distribution, mean ± SD) of patients with pCR (*n* = 11) and non-pCR (*n* = 12) before treatment were 4.9% ± 1.96% and 6.92% ± 2.22%, respectively. The frequency of Tregs before treatment in non-pCR patients was significantly higher than that in pCR patients (unpaired *t* test, *P* = 0.0317, Fig. 2A). In addition, Tregs were further subdivided into CD38 + Tregs, Ki-67 + Tregs, IFN-γ + Tregs and PD-1 + Tregs. (The gating strategy is shown in Supplementary Fig. 2A, C.) Flow cytometry analysis showed that the proportions of CD38 + Tregs, Ki-67 + Tregs, IFN-γ + Tregs and PD-1 + Tregs were not significantly different between patients with different therapeutic responses (Fig. 2B–E).

In addition, the ratios of CD8 + T cells/Tregs and PD-1 + CD8 + T cells/PD-1 + Tregs in the pCR and non-pCR groups were compared, and there was no significant difference between the two groups before treatment (Mann–Whitney test, *P* = 0.1507, Fig. 2F; unpaired *t* test, *P* = 0.1853, Fig. 2G). The receiver operating characteristic (ROC) curve was used to estimate the sensitivity and specificity, and the area under the curve (AUC) was used to estimate the predictive capacity of four indicators. The AUCs of PD-1 + Ki-67 + CD8 + T cells, CD137 + CD8 + T cells and the Treg percentage were 0.7538 (0.5509–0.9566), 0.7424 (0.5233–0.9616) and 0.7197 (0.5098–0.9296), respectively, and their predictive capacities were better than that of tissue PD-L1 (AUC = 0.6130, 0.4021–0.8239, Fig. 2H).

### Neoadjuvant chemoimmunotherapy markedly improved CD8 + T-cell proliferation and activation, especially in pCR patients

**Fig. 3:**
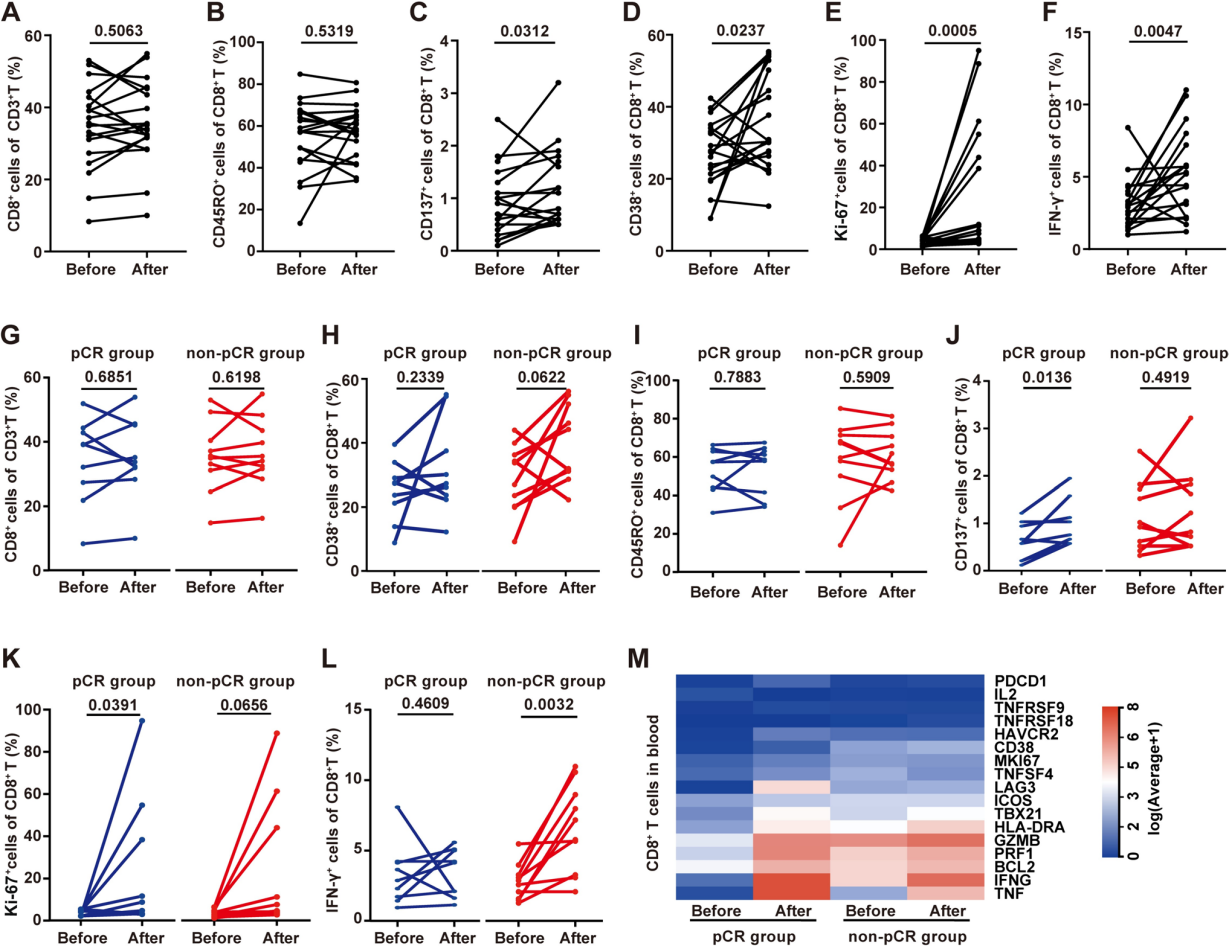
Changes in CD8 + T-cell subsets were correlated with the response to neoadjuvant chemoimmunotherapy. After 2–3 cycles of neoadjuvant chemoimmunotherapy, the changes in CD8 + T-cell subsets after treatment were analyzed, but blood samples from 19 patients were collected before surgery. **A**–**F** Comparison of the frequencies of CD8 + T cells (paired *t* test), CD45RO + CD8 + T cells (paired *t* test), CD137 + CD8 + T cells (Wilcoxon matched-pairs signed-rank test), CD38 + CD8 + T cells (paired *t* test), Ki-67 + CD8 + T cells (Wilcoxon matched-pairs signed-rank test) and IFN-γ + CD8 + T cells (Wilcoxon matched-pairs signed-rank test) before and after treatment (*n* = 19). **G**–**L** Differences in the proliferation and activation of CD8 + T-cell subsets in patients with different responses. The differences in the changes in CD8 + T-cell, CD38 + CD8 + T-cell, CD45RO + CD8 + T-cell, CD137 + CD8 + T-cell, Ki-67 + CD8 + T-cell and IFN-γ + CD8 + T-cell frequencies in pCR (*n* = 9) and non-pCR (*n* = 10) patients after treatment. **M** Comparative heatmap of the expression of CD8 + T-cell function-related genes in pCR and non-pCR patients before and after treatment

After 2–3 cycles of neoadjuvant chemoimmunotherapy, all patients underwent surgical resection of the primary tumor with lymphadenectomy and postoperative pathological evaluation. The changes in peripheral blood CD8 + T-cell subsets after treatment were analyzed, but only 19 patients’ blood samples were collected before surgery. Compared with the baseline data, we found that the CD8 + T-cell percentage (paired *t* test, *P* = 0.5063) and CD45RO + CD8 + T-cell percentage (paired *t* test, *P* = 0.5319) showed an increasing trend, but the difference did not reach statistical significance (Fig. 3A, B). On the other hand, the CD137 + CD8 + T-cell percentage changed from 0.7% ± 0.9% to 0.8% ± 1.1% (Wilcoxon matched-pairs signed-rank test, *P* = 0.0312), the percentage of CD38 + CD8 + T cells changed from 26.6% ± 9.71% to 35.56% ± 13.12% (paired *t* test, *P* = 0.0237), the percentage of Ki-67 + CD8 + T cells changed from 4% ± 2.4% to 7.5% ± 40.4% (Wilcoxon matched-pairs signed-rank test, *P* = 0.0005), and the percentage of IFN-γ + CD8 + T cells changed from 2.9% ± 2.4% to 5.2% ± 5.0% (Wilcoxon matched-pairs signed-rank test, *P* = 0.0047). These CD8 + T-cell subsets were significantly increased after treatment (Fig. 3C–F).

In addition, we analyzed whether there were differences in the proliferation and activation of CD8 + T-cell subsets in patients with different responses. We found that CD8 + T-cell, CD38 + CD8 + T-cell and CD45RO + CD8 + T-cell subsets were not significantly different between the two groups (Fig. 3G–I), but the changes in CD137 + CD8 + T-cell, Ki-67 + CD8 + T-cell and IFN-γ + CD8 + T-cell subsets were significantly different between patients with different treatment responses (Fig. 3J–L). The CD137 + CD8 + T-cell percentage of pCR patients changed from 0.64% ± 0.43% to 1.04% ± 0.54%, which was significantly increased after treatment (*n* = 9, paired *t* test, *P* = 0.0136, Fig. 3J left panel), but there was no significant change in non-pCR patients (*n* = 10, paired *t* test, *P* = 0.492, Fig. 3J right panel). The Ki-67 + CD8 + T-cell percentage of pCR patients was significantly increased from 4.3% ± 2.8% to 9% ± 43.35% after treatment (Wilcoxon matched-pairs signed-rank test, *P* = 0.0391, Fig. 3K left panel); moreover, in the non-pCR group, the mean percentage showed an increasing trend, but the difference was not significant (paired *t* test, *P* = 0.0656, Fig. 3K right panel). The median percentage of IFN-γ + CD8 + T cells in pCR patients changed from 3.0% ± 2.7% to 4.3% ± 3.3%, which was not significantly increased (Wilcoxon matched-pairs signed-rank test, *P* = 0.4609, Fig. 3L left panel); however, there was a significant increase in non-pCR patients, as the mean frequency changed from 2.79% ± 1.29% to 6.58% ± 3.13% (paired *t* test, *P* = 0.0032, Fig. 3L right panel). Furthermore, peripheral blood CD8 + T cells from patients with significantly different therapeutic responses (pCR, *n* = 2; non-MPR, *n* = 3) were sorted, and mRNA was sequenced. CD8 + T-cell proliferation, activation, exhaustion, apoptosis and function-related genes and their changes before and after treatment in pCR and non-pCR patients are shown in Fig. 3M.

### Changes in peripheral blood Treg subsets and their relationship with treatment response

**Fig. 4:**
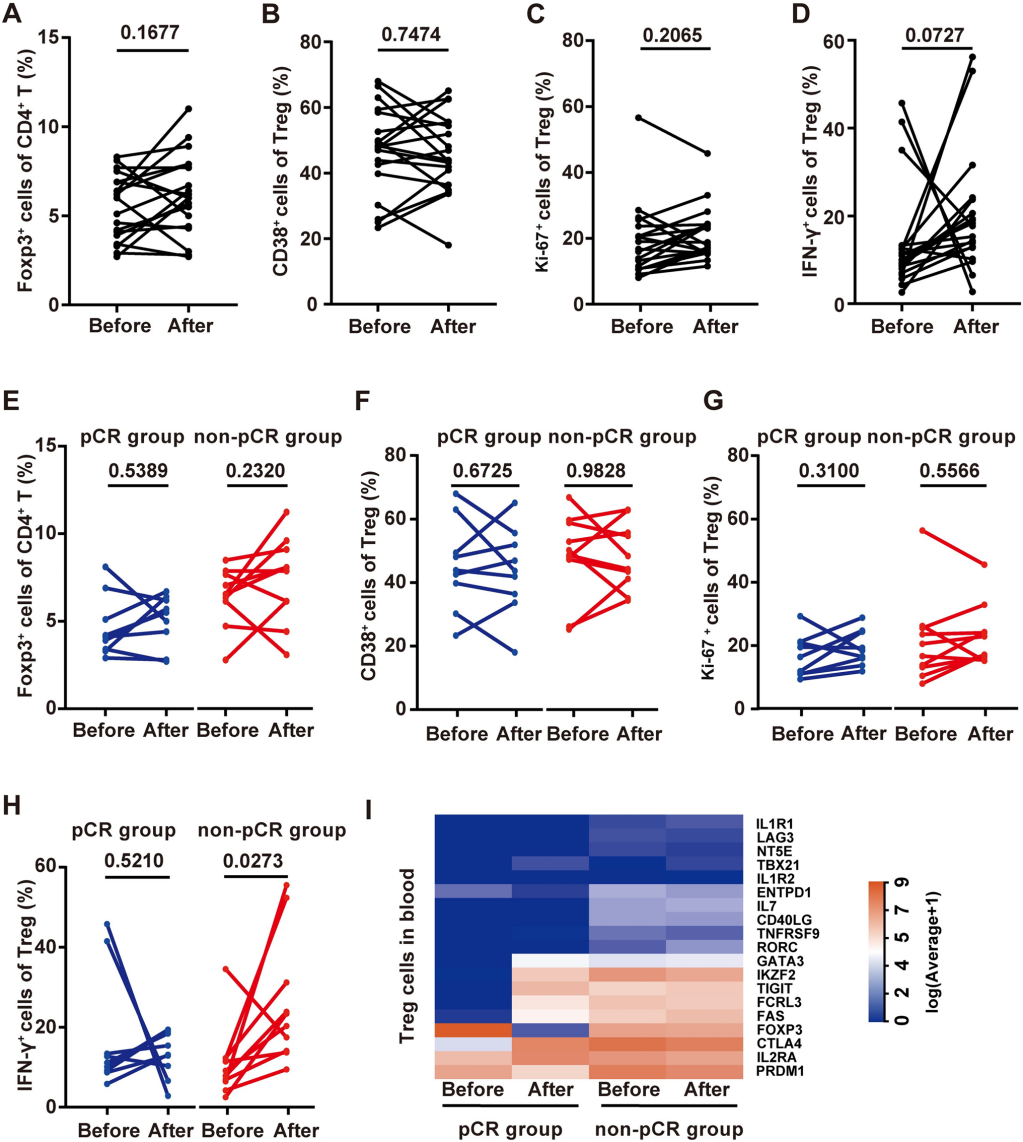
Changes in Treg subsets during neoadjuvant therapy and their relationship with neoadjuvant therapy response. **A**–**D** Changes in the Treg percentage (paired *t* test), CD38 + Treg percentage (paired *t* test), Ki-67 + Treg percentage (Wilcoxon matched-pairs signed-rank test) and IFN-γ + Treg percentage (Wilcoxon matched-pairs signed-rank test) after treatment (*n* = 19). **E**–**H** The difference in the change in the Treg percentage, CD38 + Treg percentage, Ki-67 + Treg percentage and IFN-γ + Treg percentage between pCR (*n* = 9) and non-pCR (*n* = 10) patients after treatment. I, Comparative heatmap of the expression of Treg-associated markers, including transcription factors, in pCR and non-pCR patients before and after treatment

We also analyzed the changes in Treg subsets after treatment. All Treg subsets showed an increasing trend, but the difference did not reach statistical significance (Fig. 4A–D). Similarly, the changes in Treg subsets between patients with different results were also analyzed, and there were no differences in the changes in the Treg, CD38 + Treg and Ki-67 + Treg percentages between different therapeutic response groups (Fig. 4E–G). However, the mean IFN-γ + Treg percentage changed from 17.49% ± 14.98% to 13.01% ± 5.76% in pCR patients (paired *t* test, *P* = 0.5210) and 8.65% ± 6.41% to 22.15% ± 22.75% in non-pCR patients (Wilcoxon matched-pairs signed-rank test, *P* = 0.0273), and the change trend differed significantly between the two groups (Fig. 4H). Peripheral blood Tregs from patients with significant treatment responses (pCR, *n* = 2; non-MPR, *n* = 2) were isolated, and mRNA was sequenced. The heatmap of some Treg function-related genes and transcription factors and their changes were analyzed at the mRNA level (Fig. 4I).

### Changes in cytokines in peripheral blood are indicative of treatment response

**Fig. 5:**
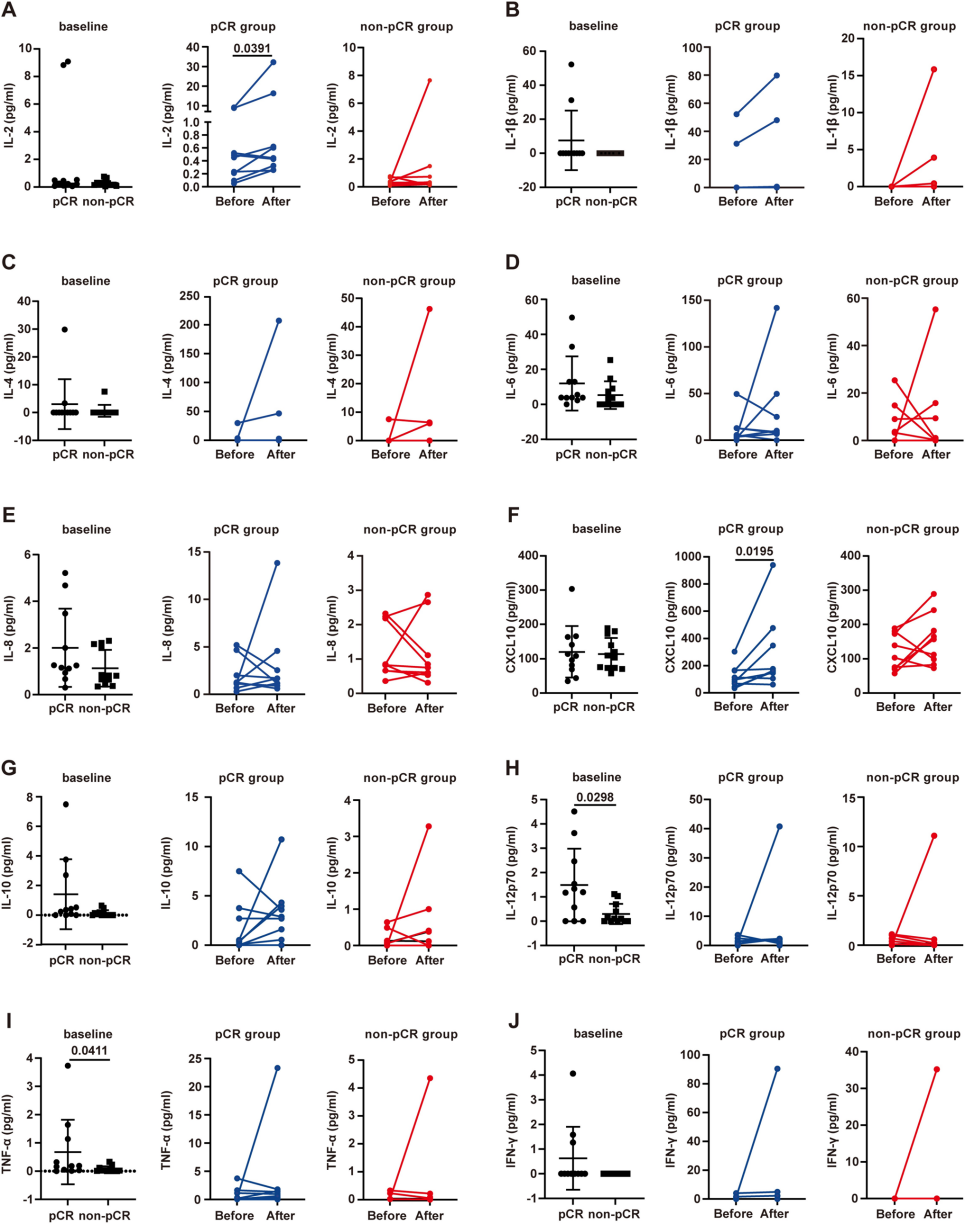
Cytokine levels and their changes reflect the response to neoadjuvant chemoimmunotherapy. The levels of cytokines in the peripheral blood of 23 patients were detected by flow cytometry before and after neoadjuvant chemoimmunotherapy, and the results are reported as the concentration (pg/mL). **A–J** Comparison of plasma IL-2, CXCL10, IL-1β, IL-10, IL-4, IL-12p70, IL-6, TNF-ɑ, IL-8 and IFN-γ levels in pCR and non-pCR patients before (*n* = 23) and after (*n* = 19) treatment

We detected the blood levels of cytokines, such as IL-1β, IL-2, IL-4, IL-6, IL-8, IL-10, IL-12p70, CXCL10 (IP-10), TNF-ɑ and IFN-γ, before and after neoadjuvant chemoimmunotherapy and analyzed the difference in cytokine levels and their change trend between different therapeutic response groups (Fig. 5A–J). Although blood IL-2 and CXCL10 levels were not different between the pCR and non-pCR groups before treatment (Fig. 5A, F left panel), the levels of these two cytokines in the pCR group increased significantly after treatment (*P* = 0.0391 and *P* = 0.0195, Fig. 5A, F middle panel). However, in the non-pCR group, blood IL-2 (*P* = 0.0742) and CXCL10 (*P* = 0.1641) levels did not increase significantly (Fig. 5A, F right panel). IL-12p70 and TNF-α were significantly different between the pCR and non-pCR groups before treatment (Fig. 5H, I left panel). There were no differences in the other cytokines (Fig. 5).

### More cytotoxic T cells were recruited to tumor sites in patients with a better response

**Fig. 6:**
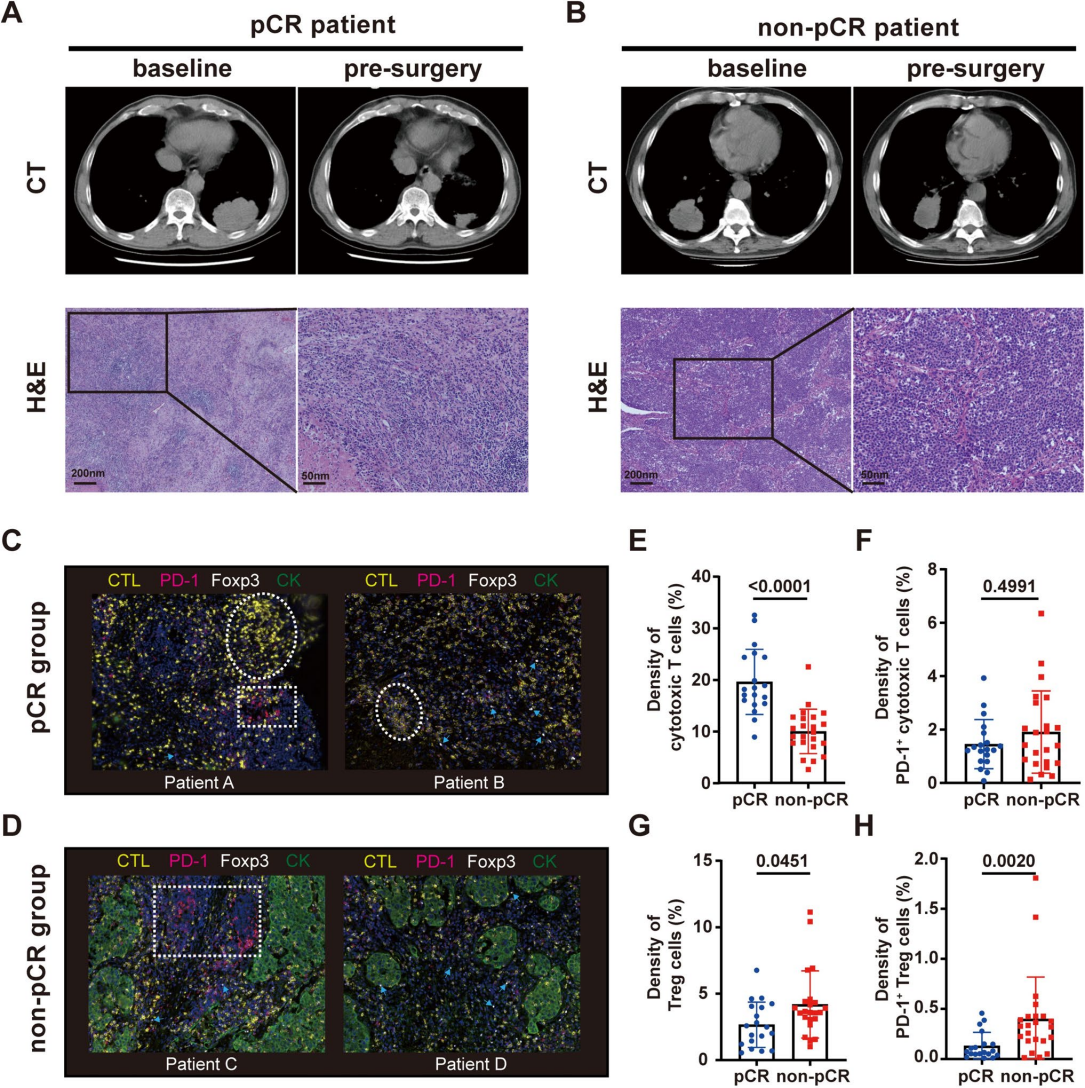
Patients with different pathological evaluations had different levels of T-subset infiltration in resected tissues after therapy. **A**, **B** Tumor size changes and tissue composition and structure differences in pCR (*n* = 19) and non-pCR (*n* = 23) patients after neoadjuvant chemoimmunotherapy. **C**, **D** Representative multiplexed images of resected tissues from pCR and non-pCR patients after neoadjuvant therapy. CK—green, cytotoxic T cells—yellow, PD-1 + cells—red, Foxp3 + cells—white (indicated by blue arrows). Cytotoxic T-cell dense regions (white dashed circle), PD-1 + cell dense region (white dashed rectangle). **E**–**H** Comparison of the density of cytotoxic T cells (Mann–Whitney test), PD-1 + cytotoxic T cells (Mann–Whitney test), Tregs (Mann–Whitney test) and PD-1 + Tregs (Mann–Whitney test) in surgical resection tissues from pCR and non-pCR patients

To evaluate the effects of neoadjuvant chemoimmunotherapy on T-cell infiltration in NSCLC and explore the mechanisms underlying why different patients had different pathological responses to neoadjuvant chemoimmunotherapy, we performed multiplex immunofluorescence to analyze the density of cytotoxic T cells, PD-1 + cytotoxic T cells, Tregs and PD-1 + Tregs in resection tissues from 42 patients. First, CT and H&E images showed the changes and differences in tumor size and tissue composition and structure of the tumor site after neoadjuvant chemoimmunotherapy in pCR and non-pCR patients (Fig. 6A–B). The multiplex immunofluorescence results showed that there were no living tumor cells in the tissues from pCR patients, and residual tumor tissue was intertwined with immune cells in resected tissues from non-MPR patients (Fig. 6C, D). Regarding the quantification of T-cell density in resection lesion samples, necrotic regions were excluded; for the non-MPR group, analyses were performed in tumor regions, which consisted of tumor cells and stroma (Fig. 6D). For MPR patients, analyses were performed in both tumor and non-tumor regions. The density of cytotoxic T cells was much higher in pCR patients than in non-pCR patients (18.67% ± 9.25% vs. 10.12% ± 5.19%, Mann–Whitney test, *P* < 0.0001, Fig. 6E). In addition, the densities of Tregs (2.49% ± 2.85% vs. 3.59% ± 1.36%) and PD-1 + Tregs (0.0725% ± 0.1025% vs. 0.3275% ± 0.2475%) were much lower in pCR patients than in non-pCR patients (Mann–Whitney test, *P* = 0.0451 and *P* = 0.002, Fig. 6G, H). There was no significant difference in the density of PD-1 + cytotoxic T cells in the pCR and non-pCR groups (1.25% ± 1.06% vs. 1.415% ± 2.255%, Mann–Whitney test, *P* = 0.4991, Fig. 6F).

## Discussion

Clinical trials such as NADIM II (NCT03838159) and CheckMate-816 have demonstrated that in patients with resectable NSCLC, neoadjuvant chemoimmunotherapy could increase the pCR and MPR rates, increase the percentage of patients suitable for definitive surgery, and extend event-free survival while not increasing the incidence of adverse events. Other key outcomes, including OS, time to death or distant metastases, objective response and radiographic downstaging, also favored chemoimmunotherapy compared with chemotherapy or ICI alone [8, 23]. In our study, the MPR rate of patients reached 62% (26/42), and pCR was achieved in 45% (19/42) of patients after treatment, which was also higher than that of chemotherapy or ICI neoadjuvant therapy, such as the neoadjuvant chemotherapy group of CheckMate -816 (37% of MPR, 2% of pCR) and CheckMate-159 (45% of MPR, 15% of pCR) [8, 24]. However, our data were slightly different from those of NADIM II (NCT03838159) and CheckMate-816, possibly due to the high proportion of squamous cell carcinoma (81%, 34/42) cases in our patient population, differences in ICI drugs or different races. According to our treatment experience, squamous cell carcinoma seems to show a better therapeutic response than adenocarcinoma or other types of NSCLC. Surgery-related indicators, such as the surgical method, operation time, blood loss, minimally invasive rate and days in the ICU, are used to evaluate the safety and feasibility of surgical treatment [25–28]. In our study, R0 resection was achieved in all patients; 61% of patients were able to undergo minimally invasive tumor resection after neoadjuvant chemoimmunotherapy, and other indicators (summarized in Table 2) were similar to those in previously reported studies [29–31]. These data suggest that neoadjuvant chemoimmunotherapy at our hospital did not significantly increase the complexity or difficulty of the operation.

Although current expert consensuses such as the *International Expert Consensus on Neoadjuvant Immunotherapy for Non-small Cell Lung Cancer* and *Expert Consensus on Perioperative Immunotherapy for Advanced Non-small Cell Lung Cancer* provide a certain guiding effect for neoadjuvant chemoimmunotherapy in clinical practice, there are still many problems that need further research, such as the treatment plan, optimal number of treatment cycles, alternative endpoints in neoadjuvant immunotherapy studies and reliable predictive markers. Not all patients benefit from neoadjuvant chemoimmunotherapy, and reliable predictive markers can be used to screen for actual beneficiaries and prevent excessive treatment for non-beneficiaries [32, 33]. Thus, the identification of reliable predictive factors is necessary.

Tissue PD-L1 expression is one of the most important predictive markers [3]. Regarding neoadjuvant immunotherapy, different studies have reported different predictive values [8, 23, 24, 34]. In our study, PD-L1 expression was not correlated with pCR (Table 1 and Supplementary Fig. 3A) or MPR (Supplementary Fig. 3B), and pCR could be achieved in patients with negative PD-L1 expression with neoadjuvant chemoimmunotherapy. Therefore, the predictive value of PD-L1 remains to be further studied.

Relevant studies have explored the potential therapeutic predictive value of circulating immune cells. For example, the frequencies of circulating NK cells, CD4 + T cells, CD4 + T-cell subsets, CD8 + T cells, CD8 + T-cell subsets, PD1 + Ki-67 + CD8 + T cells and the TCR repertoire were found to be predictive of the outcome of anti-PD-1 treatment in NSCLC patients [35–40]. However, few studies have investigated the predictive role of circulating immune cells and immune molecules in neoadjuvant chemoimmunotherapy. Our study showed that the frequency of CD137 + CD8 + T cells significantly differed between pCR and non-pCR patients, and patients with a better response had a lower CD137 + CD8 + T-cell percentage at baseline. CD137 is highly restricted to recently activated cytotoxic T cells, and CD137 + T cells at the tumor site express tumor neoantigen-reactive TCRs and are coexpressed with exhaustion markers [41–47]. Moreover, our previous study found that the percentages of CD137 + CD8 + and PD-1 + CD137 + CD8 + T-cell subsets were positively correlated with the thoracic tumor burden [48]. The high frequency of CD137 + CD8 + T cells before treatment may indicate that patients had more tumor antigen-specific, highly inhibited, exhausted CD8 + T cells and a poor response to neoadjuvant chemoimmunotherapy. We also found that pCR patients had a lower frequency of PD-1 + Ki-67 + CD8 + T cells and Tregs. Sanmamed MF et al. [49] found that CD8 + T cells with high expression of Ki-67 and PD-1 were apoptotic and dysfunctional CD8 + T cells and that their abundance was associated with tumor progression and resistance to anti-PD therapy in NSCLC patients. A high frequency of Tregs might indicate the immunosuppression status of patients. These circulating T-cell subsets showed better predictive effects than tissue PD-L1 expression (Fig. 2H).

In this study, we also found that peripheral blood CD137 + CD8 + T cells and Ki-67 + CD8 + T cells were significantly increased in pCR patients after neoadjuvant treatment. Signaling through CD137 induces the activation of CD8 + T cells in a CD28-independent manner, enhancing CD8 + T-cell survival, promoting their effector function in cancer and favoring memory CD8 + T-cell differentiation [48, 50], so the increase in these subsets may indicate that compared with patients with a poor response to treatment, in patients with a better response to treatment, CD8 + T cells can be significantly activated after treatment in terms of both quantity and ability and thus can effectively mediate the tumor-specific immune response.

Interestingly, we also found that IFN-γ + CD8 + T cells and IFN-γ + Tregs were significantly elevated after neoadjuvant chemoimmunotherapy in non-pCR patients but not in pCR patients. Indeed, early studies established IFN-γ as an antitumor cytokine; however, this cytokine was found to play a dual role in shaping the outcome of cancer [51]. On the one hand, IFN-γ from lymphocytes induces PD-L1 expression, suppressing the effector of tumor-specific T cells or NK cells and favoring cancer cell immune evasion and progression [52–55]; on the other hand, IFN-γ + Tregs are epitope-specific Tregs that suppress the proliferation of cognate epitope-specific effector cells, and the suppression was 20–50-fold greater if Tregs were specific for an epitope [56]. Lung tumor growth or some cytokines, such as IFN-γ, induce Tregs polarization to a TH1-like state, and Tregs then increase the expression of CXCR3, IFN-γ and T-bet and suppress DC1s, thereby restraining CTL activation and inducing CTL dysfunction, which might result in CTLs not infiltrating into the tumor microenvironment. In lung cancer patients, Tregs Th1 polarization correlate with poor responses to checkpoint blockade immunotherapy, and IFN-γ blockade can repolarize Tregs and restore CTLs against lung cancer [57].

Infiltrating immune cells in tumor tissues have also been a hot topic [58, 59]. By performing a retrospective analysis of the status of TILs in patients with different therapeutic responses, we found that after treatment, the densities of cytotoxic T cells, Tregs and PD-1 + Tregs were all significantly different in the resection tissues of patients in the pCR group and non-pCR group. We also found a significant increase in IL-2 and CXCL10 levels in the pCR group after treatment. IL-2 has been shown to be an essential factor for T-cell growth, proliferation and CTL and Tm production [60]. Previous studies have demonstrated that the chemokine CXCL10 plays a key role in the recruitment of CD8 + T cells to tumor sites, and increased CXCL10 levels are associated with increased tumor infiltration of CD8 + T cells and tumor clearance or regression [61–63]. The increase in CD137 + CD8 + T and Ki-67 + CD8 + T-cell frequencies and IL-2 and CXCL10 levels in peripheral blood and the enhancement of CD8 + T-cell function and increase in CD8 + T-cell density at tumor sites after neoadjuvant therapy may indicate an obvious activation of local and systemic tumor immunity, which may be the mechanisms by which patients with pCR could clear tumor cells.

In addition, we found that baseline IL-12p70 and TNF-α levels were significantly higher in the pCR group than in the non-pCR group. IL-12p70 is a proinflammatory cytokine that is involved in the link between innate and adaptive immunity [64]. Moreover, TNF-α, as a pleiotropic cytokine, plays a key role in tumor immunity and inflammation [65, 66]. Therefore, patients’ higher baseline IL-12p70 and TNF-α levels may reflect their better immune status before treatment.

Nevertheless, the sample size included in this study was small, and we hope to verify the predictive value of peripheral blood immune cell subsets and cytokines in a larger multicenter cohort of patients in the future and perform a 5-year follow-up of patients’ OS after neoadjuvant chemoimmunotherapy. In addition, patients’ tissue PD-L1 expression in this study has no correlation with the efficacy of neoadjuvant therapy, and we will continue to perform follow-up analyses.

### Supplementary Information

Below is the link to the electronic supplementary material.

Supplementary file1 (JPG 1209 KB)

Supplementary file2 (JPG 2444 KB)

Supplementary file3 (JPG 87 KB)

Supplementary file4 (DOC 110 KB)

Supplementary file5 (DOC 90 KB)

Supplementary file6 (DOC 14 KB)

## Data Availability

The raw sequence data reported in this paper have been deposited in the National Genomics Data Center, China National Center for Bioinformation/Beijing Institute of Genomics, Chinese Academy of Sciences (GSA-Human: HRA004656), which are publicly accessible at https://ngdc.cncb.ac.cn/gsa-human. Other datasets used and/or analyzed during the current study are available from the corresponding author upon reasonable request.
