# Supplementary figures and images for: T-cell subsets and cytokines are indicative of neoadjuvant chemoimmunotherapy responses in NSCLC
Source: Cancer Immunol Immunother. 2024 Apr 15;73(6):99. doi: 10.1007/s00262-024-03687-5 (PMC11018727; doi:10.1007/s00262-024-03687-5)

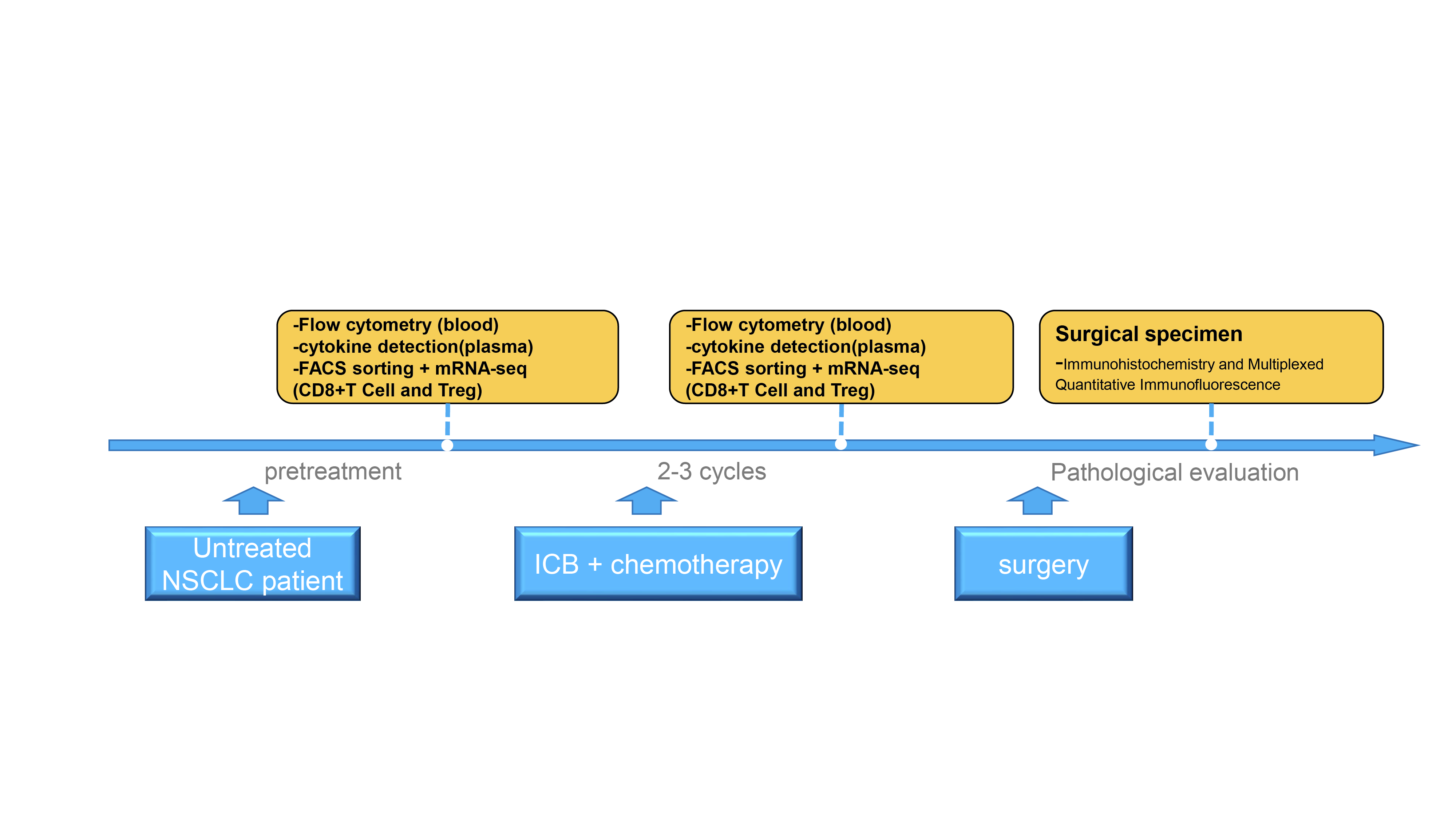

Supplement: Supplementary file 1 — Supplementary file1 (JPG 1209 KB) [file 262_2024_3687_MOESM1_ESM.jpg]

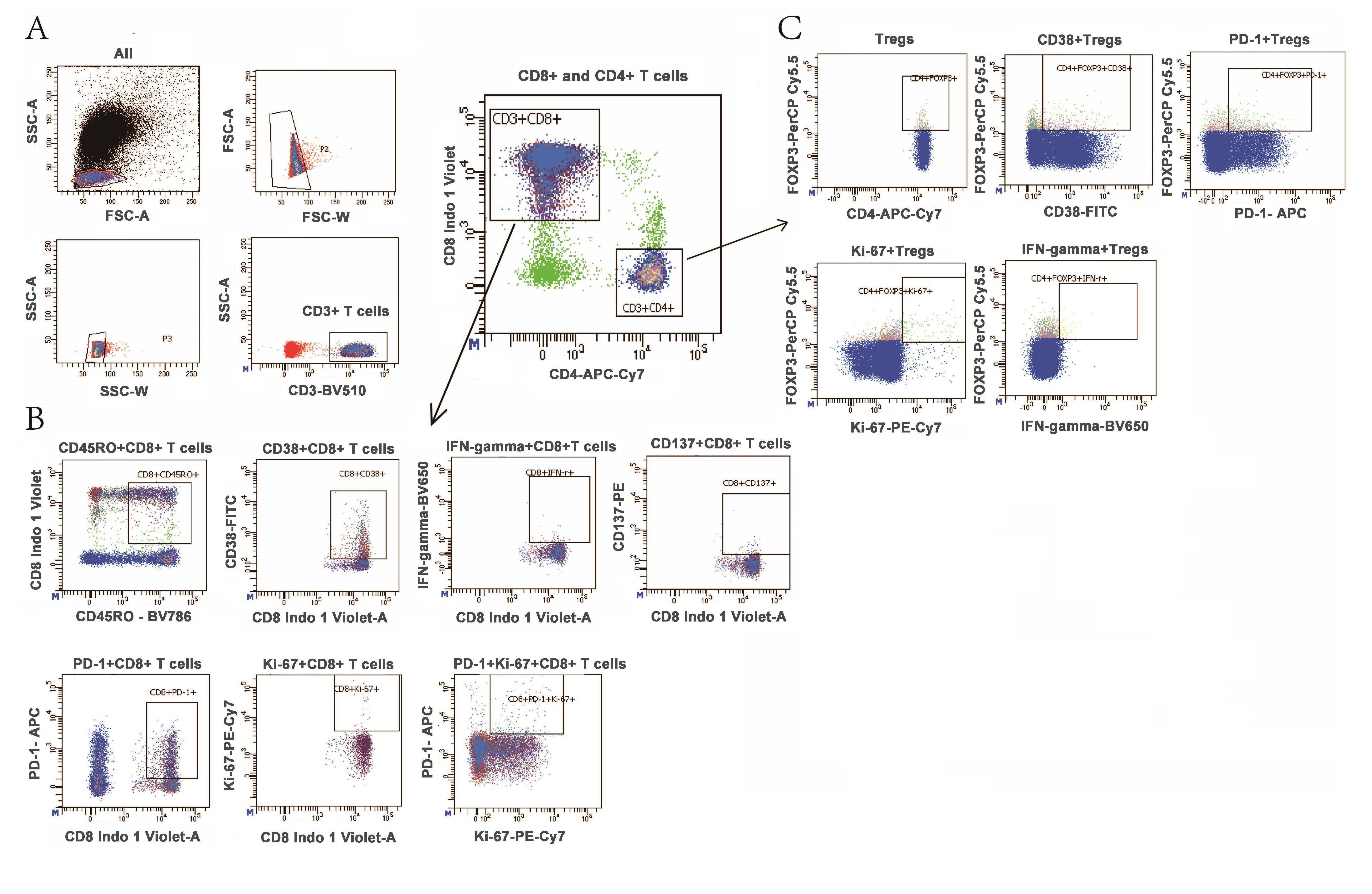

Supplement: Supplementary file 2 — Supplementary file2 (JPG 2444 KB) [file 262_2024_3687_MOESM2_ESM.jpg]

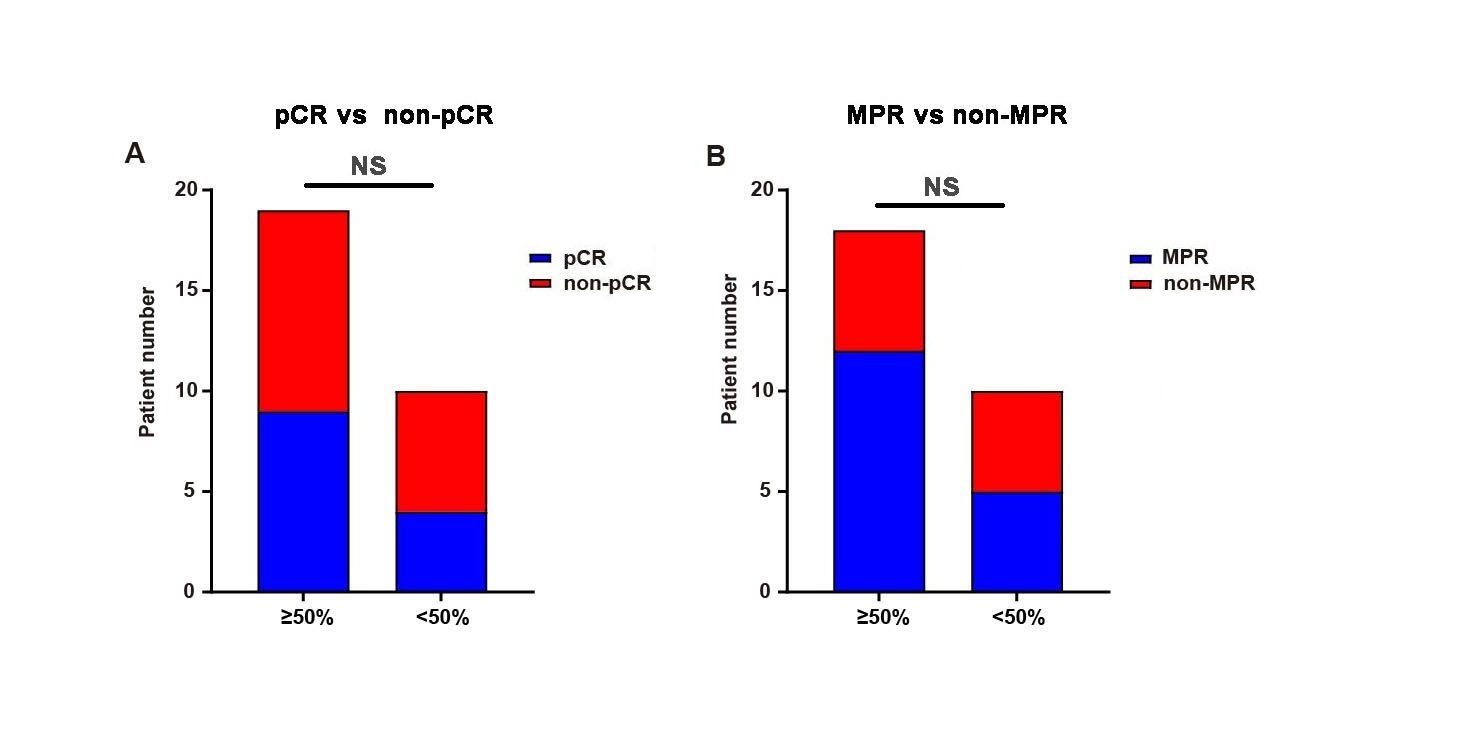

Supplement: Supplementary file 3 — Supplementary file3 (JPG 87 KB) [file 262_2024_3687_MOESM3_ESM.jpg]
