# Supplementary material for: T-cell subsets and cytokines are indicative of neoadjuvant chemoimmunotherapy responses in NSCLC
Source: Cancer Immunol Immunother. 2024 Apr 15;73(6):99. doi: 10.1007/s00262-024-03687-5 (PMC11018727; doi:10.1007/s00262-024-03687-5)
Supplement: Supplementary file 4 — Supplementary file4 (DOC 110 KB) [file 262_2024_3687_MOESM4_ESM.doc]

**Supplementary Table 1 Clinical data for 42 patients**

| Patient | Sex | Age | Histology | Clinical stage | Therapeutic schedule | Chemotherapeutic drugs | Prescribed checkpoint inhibitor | Neoadjuvant therapy cycles | Surgical approach | Pathological response | RECIST status |
| --- | --- | --- | --- | --- | --- | --- | --- | --- | --- | --- | --- |
| Patient1 | Male | 58 | lung squamous carcinoma | IIIA | paclitaxel liposome+carboplatin+tislelizumab | paclitaxel liposome+carboplatin | tislelizumab | 2 | pulmonary lobectomy | MPR | PR |
| Patient2 | Male | 67 | lung squamous carcinoma | IIB | albumin paclitaxel+carboplatin+camrelizumab | albumin paclitaxel+carboplatin | camrelizumab | 2 | pulmonary lobectomy | pCR | PR |
| Patient3 | Male | 73 | lung squamous carcinoma | IIB | albumin paclitaxel+carboplatin+camrelizumab | albumin paclitaxel+carboplatin | camrelizumab | 2 | sleeve resection | MPR | PR |
| Patient4 | Male | 58 | lung squamous carcinoma | IIB | albumin paclitaxel+carboplatin+camrelizumab | albumin paclitaxel+carboplatin | camrelizumab | 2 | pulmonary lobectomy | pCR | PR |
| Patient5 | Male | 73 | lung adenocarcinoma | IIIB | paclitaxel liposome+carboplatin+pembrolizumab | paclitaxel liposome+carboplatin | pembrolizumab | 2 | pulmonary lobectomy | pCR | PR |
| Patient6 | Female | 66 | lung squamous carcinoma | IIIA | albumin paclitaxel+carboplatin+sintilimab | albumin paclitaxel+carboplatin | sintilimab | 2 | sleeve resection | pCR | PR |
| Patient7 | Female | 58 | lung adenocarcinoma | IIIA | carboplatin+pemetrexed+camrelizumab | carboplatin+pemetrexed | camrelizumab | 2 | pulmonary lobectomy | MPR | PR |
| Patient8 | Male | 67 | lung squamous carcinoma | IIIB | albumin paclitaxel+carboplatin+sintilimab | albumin paclitaxel+carboplatin | sintilimab | 2 | pulmonary lobectomy | ＜MPR | SD |
| Patient9 | Male | 72 | lung squamous carcinoma | IIIB | paclitaxel liposome+carboplatin+tislelizumab | paclitaxel liposome+carboplatin | tislelizumab | 2 | pulmonary lobectomy | ＜MPR | PR |
| Patient10 | Male | 65 | lung squamous carcinoma | IIIA | albumin paclitaxel+carboplatin+camrelizumab | albumin paclitaxel+carboplatin | camrelizumab | 2 | pulmonary lobectomy | ＜MPR | SD |
| Patient11 | Male | 65 | lung squamous carcinoma | IIIB | albumin paclitaxel+carboplatin+sintilimab | albumin paclitaxel+carboplatin | sintilimab | 2 | pulmonary lobectomy | ＜MPR | SD |
| Patient12 | Male | 75 | lung squamous carcinoma | IIIA | albumin paclitaxel+carboplatin+tislelizumab | albumin paclitaxel+carboplatin | tislelizumab | 2 | pulmonary lobectomy | ＜MPR | PR |
| Patient13 | Male | 58 | lung squamous carcinoma | IIIB | gemcitabine+nedaplatin+sintilimab | gemcitabine+nedaplatin | sintilimab | 2 | pulmonary lobectomy | pCR | PR |
| Patient14 | Male | 66 | lung squamous carcinoma | IIIB | paclitaxel liposome+carboplatin+sintilimab | paclitaxel liposome+carboplatin | sintilimab | 2 | pulmonary lobectomy | ＜MPR | PR |
| Patient15 | Male | 59 | lung squamous carcinoma | IIIA | albumin paclitaxel+carboplatin+camrelizumab | albumin paclitaxel+carboplatin | camrelizumab | 2 | pulmonary lobectomy | pCR | PR |
| Patient16 | Male | 72 | lung adenocarcinoma | IIIA | albumin paclitaxel+carboplatin+sintilimab | albumin paclitaxel+carboplatin | sintilimab | 3 | pulmonary lobectomy | ＜MPR | PR |
| Patient17 | Male | 73 | lung squamous carcinoma | IIIB | albumin paclitaxel+carboplatin+camrelizumab | albumin paclitaxel+carboplatin | camrelizumab | 2 | pulmonary lobectomy | pCR | PR |
| Patient18 | Male | 68 | lung squamous carcinoma | IIIA | albumin paclitaxel+carboplatin+camrelizumab | albumin paclitaxel+carboplatin | camrelizumab | 2 | bilobar resection | pCR | PR |
| Patient19 | Female | 59 | lung adenocarcinoma | IIIA | pemetrexed+carboplatin+sintilimab | pemetrexed+carboplatin | sintilimab | 3 | pulmonary lobectomy | ＜MPR | PR |
| Patient20 | Male | 58 | lung squamous carcinoma | IIIB | albumin paclitaxel+carboplatin+tislelizumab | albumin paclitaxel+carboplatin | tislelizumab | 2 | pulmonary lobectomy | ＜MPR | PR |
| Patient21 | Male | 56 | lung adenocarcinoma | IIIB | carboplatin+pemetrexed+sintilimab | carboplatin+pemetrexed | sintilimab | 3 | pulmonary lobectomy | pCR | PR |
| Patient22 | Male | 69 | lung squamous carcinoma | IIIB | albumin paclitaxel+carboplatin+camrelizumab | albumin paclitaxel+carboplatin | camrelizumab | 2 | pulmonary lobectomy | pCR | SD |
| Patient23 | Male | 67 | lung squamous carcinoma | IIIA | albumin paclitaxel+carboplatin+camrelizumab | albumin paclitaxel+carboplatin | camrelizumab | 2 | pulmonary lobectomy | pCR | PR |
| Patient24 | Male | 49 | lung adenocarcinoma | IIB | albumin paclitaxel+carboplatin+camrelizumab | albumin paclitaxel+carboplatin | camrelizumab | 2 | pulmonary lobectomy | pCR | SD |
| Patient25 | Male | 70 | lung squamous carcinoma | IIIB | albumin paclitaxel+carboplatin+camrelizumab | albumin paclitaxel+carboplatin | camrelizumab | 2 | pulmonary lobectomy | pCR | PR |
| Patient26 | Male | 59 | lung squamous carcinoma | IIB | albumin paclitaxel+carboplatin+camrelizumab | albumin paclitaxel+carboplatin | camrelizumab | 2 | pulmonary lobectomy | pCR | SD |
| Patient27 | Male | 52 | lung squamous carcinoma | IIB | albumin paclitaxel+carboplatin+camrelizumab | albumin paclitaxel+carboplatin | camrelizumab | 2 | sleeve resection | pCR | SD |
| Patient28 | Male | 66 | lung squamous carcinoma | IIB | albumin paclitaxel+carboplatin+camrelizumab | albumin paclitaxel+carboplatin | camrelizumab | 2 | sleeve resection | pCR | SD |
| Patient29 | Male | 63 | lung squamous carcinoma | IIIA | albumin paclitaxel+carboplatin+camrelizumab | albumin paclitaxel+carboplatin | camrelizumab | 2 | bilobar resection | pCR | SD |
| Patient30 | Male | 60 | lung squamous carcinoma | IIB | albumin paclitaxel+carboplatin+camrelizumab | albumin paclitaxel+carboplatin | camrelizumab | 2 | pulmonary lobectomy | pCR | PR |
| Patient31 | Male | 60 | lung squamous carcinoma | IIIA | albumin paclitaxel+carboplatin+camrelizumab | albumin paclitaxel+carboplatin | camrelizumab | 2 | sleeve resection | pCR | PR |
| Patient32 | Male | 67 | lung squamous carcinoma | IIIB | albumin paclitaxel+carboplatin+camrelizumab | albumin paclitaxel+carboplatin | camrelizumab | 2 | bilobar resection | MPR | SD |
| Patient33 | Male | 72 | lung squamous carcinoma | IIB | albumin paclitaxel+carboplatin+camrelizumab | albumin paclitaxel+carboplatin | camrelizumab | 2 | pulmonary lobectomy | MPR | PR |
| Patient34 | Male | 61 | lung squamous carcinoma | IIIB | albumin paclitaxel+carboplatin+camrelizumab | albumin paclitaxel+carboplatin | camrelizumab | 2 | bilobar resection | MPR | PR |
| Patient35 | Male | 68 | lung squamous carcinoma | IIIA | albumin paclitaxel+carboplatin+camrelizumab | albumin paclitaxel+carboplatin | camrelizumab | 2 | pulmonary lobectomy | MPR | PR |
| Patient36 | Female | 68 | lung squamous carcinoma | IIB | albumin paclitaxel+carboplatin+camrelizumab | albumin paclitaxel+carboplatin | camrelizumab | 2 | pulmonary lobectomy | ＜MPR | SD |
| Patient37 | Male | 55 | lung adenocarcinoma | IIIA | albumin paclitaxel+carboplatin+camrelizumab | albumin paclitaxel+carboplatin | camrelizumab | 2 | bilobar resection | ＜MPR | SD |
| Patient38 | Male | 57 | lung squamous carcinoma | IIA | albumin paclitaxel+carboplatin+camrelizumab | albumin paclitaxel+carboplatin | camrelizumab | 2 | pulmonary lobectomy | ＜MPR | SD |
| Patient39 | Male | 66 | lung squamous carcinoma | IIA | albumin paclitaxel+carboplatin+camrelizumab | albumin paclitaxel+carboplatin | camrelizumab | 2 | pulmonary lobectomy | ＜MPR | SD |
| Patient40 | Male | 61 | lung squamous carcinoma | IIIA | albumin paclitaxel+carboplatin+camrelizumab | albumin paclitaxel+carboplatin | camrelizumab | 2 | bilobar resection | ＜MPR | PR |
| Patient41 | Male | 57 | lung squamous carcinoma | IIIA | albumin paclitaxel+carboplatin+camrelizumab | albumin paclitaxel+carboplatin | camrelizumab | 2 | sleeve resection | ＜MPR | SD |
| Patient42 | Male | 66 | lung adenocarcinoma | IIIA | albumin paclitaxel+carboplatin+camrelizumab | albumin paclitaxel+carboplatin | camrelizumab | 2 | pulmonary lobectomy | ＜MPR | SD |
