# Supplementary material for: T-cell subsets and cytokines are indicative of neoadjuvant chemoimmunotherapy responses in NSCLC
Source: Cancer Immunol Immunother. 2024 Apr 15;73(6):99. doi: 10.1007/s00262-024-03687-5 (PMC11018727; doi:10.1007/s00262-024-03687-5)
Supplement: Supplementary file 5 — Supplementary file5 (DOC 90 KB) [file 262_2024_3687_MOESM5_ESM.doc]

**Supplementary Table 2 PD-L1 immunohistochemical data for 42 patients**

| Patient | PD-L1 expression | PD-L1 clonal selection | | CK7 | CKpan | TTF-1 | Napsin-A | p40 | CK5/6 | Others |
| --- | --- | --- | --- | --- | --- | --- | --- | --- | --- | --- |
| Patient1 | 70 | | 22C3 | - | + | - | - | + | + |  |
| Patient2 | 2 | | 22C3 | - | + | - | - | + | + |  |
| Patient3 | 1 | | 22C3 | - | + | - | - | + | + |  |
| Patient4 | 15 | | 22C3 | Partial+ | + | - | - | + | + |  |
| Patient5 | 95 | | 22C3 |  | + | + | - |  | - | Tg(-),PAX-8(-),CD56(-),Syn(-) |
| Patient6 | 95 | | 22C3 | - | + | - | - | + | + |  |
| Patient7 | 100 | | 22C3 | + | + | + | + | - | few+ |  |
| Patient8 | 30 | | 22C3 |  | + | - | - | + | + |  |
| Patient9 | 1 | | 22C3 |  | + | - | - | + | + |  |
| Patient10 | 65 | | 22C3 | - | + | few+ | - | + | Partial+ |  |
| Patient11 | 80 | | 22C3 | - | + | - | - | + | + |  |
| Patient12 | 90 | | 22C3 |  | + | - | - | + | + |  |
| Patient13 | 70 | | 22C3 |  | + | - | - | + | + | HER2(-) |
| Patient14 | 10 | | 22C3 |  | + | - | - | + | - |  |
| Patient15 | N/A | | N/A | N/A | N/A | N/A | N/A | N/A | N/A |  |
| Patient16 | 60 | | 22C3 | + |  | + | + | - |  |  |
| Patient17 | N/A | | N/A | N/A | N/A | N/A | N/A | N/A | N/A |  |
| Patient18 | N/A | | N/A | N/A | N/A | N/A | N/A | N/A | N/A |  |
| Patient19 | N/A | | N/A | N/A | N/A | N/A | N/A | N/A | N/A |  |
| Patient20 | 0 | | 22C3 | - | + | - | - | + | + |  |
| Patient21 | 0 | | 22C3 | + | + | - |  |  |  | CK20(-),CDX-2(-),ViLLin(-) |
| Patient22 | 40 | | 22C3 | - | + | - | - | + | + | HER2(0),CD56(-),Syn(-) |
| Patient23 | N/A | | N/A | N/A | N/A | N/A | N/A | N/A | N/A |  |
| Patient24 | 55 | | 22C3 | + | + | + | + | + | - |  |
| Patient25 | N/A | | N/A | N/A | N/A | N/A | N/A | N/A | N/A |  |
| Patient26 | 80 | | 22C3 | - | + | - | - | + | + |  |
| Patient27 | 100 | | 22C3 |  |  | - | - | + | + | HER2(2+) |
| Patient28 | 100 | | 22C3 | - | + | - | - | + | + |  |
| Patient29 | N/A | | N/A | N/A | N/A | N/A | N/A | N/A | N/A |  |
| Patient30 | 80 | | 22C3 | - | + | - | - | + | + |  |
| Patient31 | 80 | | 22C3 |  | + | - |  | - | + |  |
| Patient32 | N/A | | N/A | N/A | N/A | N/A | N/A | N/A | N/A |  |
| Patient33 | 70 | | 22C3 | + | + | - | - | + | + |  |
| Patient34 | 75 | | 22C3 |  | + | - |  | - |  |  |
| Patient35 | N/A | | N/A | N/A | N/A | N/A | N/A | N/A | N/A |  |
| Patient36 | 100 | | 22C3 | + | + | - | - | + | + |  |
| Patient37 | N/A | | N/A | + |  | + | + |  |  | CEA(+) |
| Patient38 | 0 | | 22C3 | + | + | - | - | + | + |  |
| Patient39 | N/A | | N/A | N/A | N/A | N/A | N/A | N/A | N/A |  |
| Patient40 | 60 | | 22C3 | - | + | - | - | + | + |  |
| Patient41 | N/A | | N/A | N/A | N/A | N/A | N/A | N/A | N/A |  |
| Patient42 | N/A | | N/A | + | + | + | + | - | - |  |
