# Supplementary material for: T-cell subsets and cytokines are indicative of neoadjuvant chemoimmunotherapy responses in NSCLC
Source: Cancer Immunol Immunother. 2024 Apr 15;73(6):99. doi: 10.1007/s00262-024-03687-5 (PMC11018727; doi:10.1007/s00262-024-03687-5)
Supplement: Supplementary file 6 — Supplementary file6 (DOC 14 KB) [file 262_2024_3687_MOESM6_ESM.doc]

Supplementary Table 3 Classification of T-cell subpopulations

| T-cell subsets | Activation | Proliferation | Effector | Memory | Exhaustion |
| --- | --- | --- | --- | --- | --- |
| CD8+ T | CD137, CD38 | Ki-67 | IFN-γ | CD45RO | PD-1 |
| Treg | CD38 | Ki-67 | IFN-γ |  | PD-1 |
